# County-Level Variation in Cirrhosis-Related Mortality in the US, 1999-2019

**DOI:** 10.1001/jamanetworkopen.2021.46427

**Published:** 2022-02-02

**Authors:** Audrey Y. Ha, Michael H. Le, Linda Henry, Yee Hui Yeo, Ramsey C. Cheung, Mindie H. Nguyen

**Affiliations:** 1 Division of Gastroenterology and Hepatology, Stanford University Medical Center, Palo Alto, California; 2 Department of Medicine, Cedars Sinai Medical Center, Los Angeles, California; 3 Division of Gastroenterology and Hepatology, VA Palo Alto HCS, Palo Alto, California; 4 Department of Epidemiology and Population Health, Stanford University Medical Center, Palo Alto, California

## Abstract

This cross-sectional study characterizes cirrhosis mortality by metropolitan categories and potential disparities among the various rural and urban demographic subgroups using the US Centers for Disease Control and Prevention WONDER database.

## Introduction

Cirrhosis is the end stage of chronic liver diseases with a median life expectancy of 2 years for decompensated cirrhosis.^1^ With limited resources, identifying where to target prevention efforts is critical. To date, studies of cirrhosis mortality have primarily focused on age, sex, ethnicity, and etiology.^2,3^ Therefore, we aimed to characterize cirrhosis mortality by metropolitan categories as well as potential disparities among the various rural and urban demographic subgroups.

## Methods

This cross-sectional study did not require institutional review board (IRB) approval or informed consent per institutional policy at Stanford University because the WONDER data are deidentified and publicly available. This study followed the Strengthening the Reporting of Observational Studies in Epidemiology (STROBE) reporting guideline.

Using the US Centers for Disease Control and Prevention WONDER database (1999-2019),^4^ we analyzed decedents of any age with cirrhosis as the underlying cause of death. We reported race and ethnicity and obtained age-adjusted mortality as provided by WONDER. Participant race and ethnicity were collected based on the information on the participant’s death certificate. Race and ethnicity categories were Hispanic, non-Hispanic American Indian or Alaskan Native, non-Hispanic Asian or Pacific Islander, non-Hispanic Black or African American, and non-Hispanic White. Race and ethnicity were variables considered in this study to better understand which populations are most affected by cirrhosis.

 We determined trends in age-adjusted mortality using the Joinpoint regression program, version 4.9.0 (National Cancer Institute), which summarizes the data using 1 or multiple trend line segments through piecewise linear regression.^5^ Further details on cirrhosis and metropolitan category definitions and on Joinpoint analysis are in eMethods in the Supplement. A χ^2^ test was used to calculate the *P* values for the age-adjusted mortality rates in the table. A Monte Carlo permutation method was used to calculate the *P* values for the Joinpoint models. All tests were 2-sided, and statistical significance was set at *P* < .05. Statistical analysis was performed from April 2021 to December 2021.

## Results

From 1999 to 2019, there were 701 863 deceased participants with cirrhosis: 437 342 (62.3%) were male; 61 436 (8.8%) were aged 44 years or younger, 495 857 (70.7%) were aged 45 to 74, and 144 517 (20.6%) were aged 75 years or older; 518 036 (73.8%) were non-Hispanic White individuals, 92 573 (13.2%) were Hispanic individuals, and 12 949 (1.8%) were non-Hispanic American Indian individuals. In large central metropolitan areas, 118 054 of 194 243deaths (60.8%) occurred in medical facilities compared with 37 657 of 72 590 (51.9%) in small metropolitan areas.

There were differences in age-adjusted mortality rate per 100 000 by metropolitan categories overall (large fringe metropolitan areas: 8.7; 95% CI, 8.6-8.7; small metropolitan areas: 11.1; 95% CI, 11.0-11.2), and mortality was approximately 2 times higher for men overall and in each metropolitan category (Table). In addition to disparities in age-adjusted mortality rate per 100 000 among different racial and ethnic groups, there were also large disparities by metropolitan categories within the Hispanic groups (large fringe metropolitan areas: 10.8; 95% CI, 10.6-11.0; nonmetropolitan areas: 18.0; 95% CI, 17.6-18.5) and the non-Hispanic American Indian groups (large fringe metropolitan area: 15.3; 95% CI, 14.2-16.3; nonmetropolitan area: 33.5; 95% CI, 32.6-34.3).

**Table.** Age-Adjusted Cirrhosis Mortality Rate per 100 000 People and 95% CI in the US (1999-2019)

Characteristics	No. %	Cirrhosis, rate per 100 000 people (95% CI)						
		All categories (n = 701 863)^a^	Large central (n = 194 243)^a^	Large fringe (n = 148 877)^a^	Medium (n = 158 377)^a^	Small (n = 72 590)^a^	Non (n = 127 776)^a^	*P* value
Overall		10.0 (10.0-10.0)	9.8 (9.7-9.8)	8.7 (8.6-8.7)	10.8 (10.8-10.9)	11.1 (11.0-11.2)	10.9 (10.8-11.0)	<.001
Sex								
Male	437 342 (62.3)	13.4 (13.4-13.4)	13.4 (13.3-13.5)	11.4 (11.4-11.5)	14.5 (14.4-14.6)	14.4 (14.3-14.6)	14.4 (14.3-14.5)	<.001
Female	264 521 (37.7)	7.0 (7.0-7.1)	6.6 (6.6-6.7)	6.2 (6.2-6.3)	7.6 (7.5-7.6)	7.8 (7.7-7.9)	7.9 (7.8-8.0)	<.001
Race and ethnicity								
Hispanic	92 573 (13.2)	14.4 (14.3-14.5)	13.6 (13.4-13.7)	10.8 (10.6-11.0)	17.7 (17.5-17.9)	16.7 (16.3-17.2)	18.0 (17.6-18.5)	<.001
Non-Hispanic								
American Indian	12 949 (1.8)	27.2 (26.7-27.7)	23.8 (22.8-24.9)	15.3 (14.2-16.3)	23.7 (22.7-24.7)	30.1 (28.6-31.6)	33.5 (32.6-34.3)	<.001
Asian or Pacific Islander	10 820 (1.5)	3.8 (3.7-3.8)	3.5 (3.4-3.6)	3.5 (3.3-3.6)	4.9 (4.7-5.1)	4.7 (4.2-5.1)	4.8 (4.3-5.2)	<.001
Black or African American	65 120 (9.3)	8.8 (8.7-8.9)	9.2 (9.1-9.3)	7.1 (6.9-7.2)	9.2 (9.1-9.4)	9.8 (9.5-10.0)	9.6 (9.3-9.8)	<.001
White	518 036 (73.8)	9.9 (9.9-9.9)	9.7 (9.6-9.7)	8.9 (8.9-9.0)	10.4 (10.4-10.5)	10.6 (10.5-10.7)	10.4 (10.3-10.4)	<.001

^a^ Metropolitan categories defined: large central metropolitan areas (>1 million people containing a significant percentage of principal city: ie, inner city), large fringe metropolitan areas (>1 million people but does not contain a significant percentage of principal city: ie, suburb), medium metropolitan areas (250 000-999 999 people), small metropolitan areas (50 000-249 999 people), and nonmetropolitan areas (<50 000 people).

Furthermore, while age-adjusted cirrhosis mortality increased overall and in all metropolitan categories from approximately 2008 onwards (Figure), the highest annual percentage changes (APCs) occurred in nonmetropolitan areas (2008-2019: 3.7%; 95% CI, 3.3%-4.0%), small metropolitan areas (2008-2015: 3.8%; 95% CI, 3.2%-4.5%), and medium metropolitan areas (2009-2015: 3.4%; 95% CI, 2.3%-4.5%) (all *P* < .001). The lowest APC was in large central metropolitan areas (2013-2019: 0.4%; 95% CI, 0.1-0.8%; *P* = .03). Notably, American Indian participants also had the highest APC in cirrhosis mortality (2009-2019: 3.7%; 95% CI, 3.0-4.4%, *P* < .001), while APCs for other racial and ethnic groups ranged from 0.0% to 1.9% for the most recent joinpoint segment between 1999 and 2019.

**Figure.**
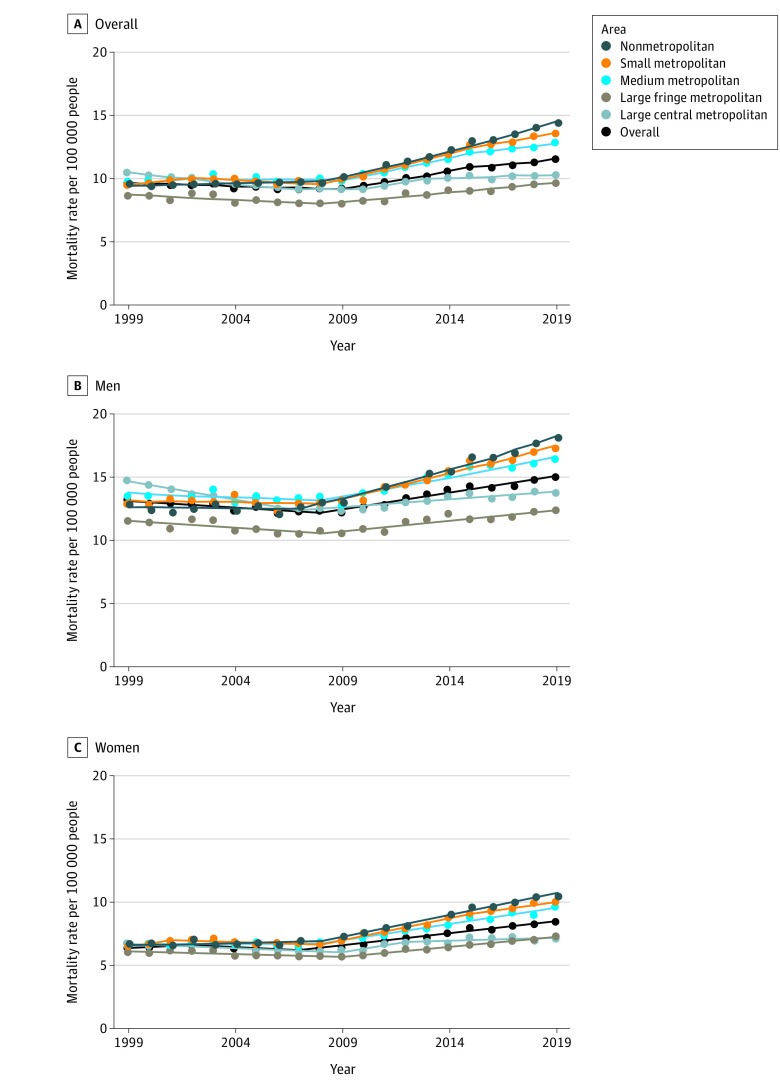
Age-Adjusted Cirrhosis Mortality Rate per 100 000 People in the United States From 1999-2019, Overall and by Sex Metropolitan categories defined: large central metropolitan areas (more than 1 million people containing a substantial percentage of principal city: ie, inner city), large fringe metropolitan areas (more than 1 million people but does not contain a substantial percentage of principal city: ie, suburb), medium metropolitan areas (250 000-999 999 people), small metropolitan areas (50 000-249 999 people), and nonmetropolitan areas (<50 000 people).

## Discussion

In this study, we observed increasing cirrhosis mortality from 2009 to 2019 in the US overall, with the highest rates in smaller towns and more rural areas. We also found the largest urban-rural disparities among the Hispanic and American Indian populations, with mortality rates approximately twice as high or higher in rural regions (ie, nonmetropolitan areas) vs suburban regions (large fringe metropolitan areas) for these groups. Thus, to address these disparities, further public health intervention and policy changes are needed to provide additional resources to rural communities, especially those communities with high concentrations of Hispanic and American Indian individuals.

Though the majority of non-Hispanic American Indian individuals live close to reservations to obtain services from the Indian Health Service and other federal programs,^6^ many still receive care through underfunded agencies, leading to worse outcomes. A significant proportion of the Hispanic population may be migrant farm workers with limited access to health care facilities.

Our study limitations include a lack of relevant socioeconomic data. Further research is necessary to determine how factors such as distance to general and subspecialty liver care, transportation resources, and health insurance coverage, contribute to the observed disparities and how these disparities can be rectified.

## Notes

Supplement.
eMethods.
