# Supplementary material for: County-Level Variation in Cirrhosis-Related Mortality in the US, 1999-2019
Source: JAMA Netw Open. 2022 Feb 2;5(2):e2146427. doi: 10.1001/jamanetworkopen.2021.46427 (PMC8811626; doi:10.1001/jamanetworkopen.2021.46427)
Supplement: Supplement. — eMethods. [file jamanetwopen-e2146427-s001.pdf]

Supplemental Online Content

Ha AY, Le MH, Henry L, Yeo YH, Cheung RC, Nguyen MH. County-level variation in cirrhosis-related mortality in the US, 1999-2019. *JAMA Netw Open*. 2022;5(2):e2146427. doi:10.1001/jamanetworkopen.2021.46427

eMethods.

This supplemental material has been provided by the authors to give readers additional information about their work.

© 2022 Ha AY et al. *JAMA Network Open.*

eMethods.

CDC WONDER is an online public health database provided by the Centers for Disease Control and Prevention, which covers more than 99% of deaths from 57 different vital statistics jurisdictions throughout the United States and its territories. Thus, the database is representative of U.S. deaths (missing data is <1%) and there is a low risk of bias. The CDC WONDER database records the cause of death as the single underlying or multiple causes of death. Underlying cause of death refers to the main disease or condition that leads to death. Decedents with cirrhosis, of any age, were identified using the ICD-10 (international classification of diseases, 10th revision) codes of G93.4, I85.0, I86.4, K70.3, K71.7, K72.9, K74.3, K74.4, K74.5, K74.6, K76.6, K76.7, and R18. From 1999-2019, there were a total of 53,422,612 deaths in the United States. 701,863 had cirrhosis as the underlying cause of death and were thus included in this study.

Metro categories were defined by the CDC's 2013 Urban-Rural Classification Scheme for Counties: large central metro (>1 million people containing a significant percentage of principal city: ie. inner city), large fringe metro (>1 million people but does not contain a significant percentage of principal city: ie. suburb), medium metro (250,000-999,999 people), small metro (50,000-249,999 people), and non-metro (<50,000 people).

CDC WONDER calculates crude mortality rates for the metro categories and relevant demographic variables by dividing the number of deaths by the population size from the U.S. Census Bureau, and then recalculating to be per 100,000 persons. In order to calculate age-adjusted mortality rates, the database applied age-specific crude mortality rates to the age distribution of the 2000 U.S. standard population. A chi square test was used to compare age-adjusted mortality rates among the different subgroups.

We determined trends in age-adjusted mortality rates using the National Cancer Institute's joinpoint regression program (version 4.9.0). Joinpoint analysis allowed us to assess if any of the years from 1999-2019 had a different rate of change with statistical significance. The joinpoint program summarized the data using one or multiple line segments, fitting the simplest joinpoint model through piecewise linear regression to create the graphs in Figures 1a-1c. A weighted least squares method was used to calculate the annual percent change (APC) for each line segment. *P*-values were calculated using a Monte Carlo Permutation method, where *P*-value < 0.05 suggests that the APC differs from zero and the difference is statistically significant.

To further examine trends related to urbanization, we conducted joinpoint analysis for the 5 metro categories within each of the following demographic groups: males, females, Hispanic individuals, non-Hispanic American Indian individuals, non-Hispanic Asian individuals and Pacific Islander individuals, non-Hispanic Black and African American individuals, and non-Hispanic White individuals. R programming (version 4.0.2) was used for chi square analysis.

© 2022 Ha AY et al. *JAMA Network Open.*
